# Combination of C-X-C motif chemokine 9 and C-X-C motif chemokine 10 antibodies with FTY720 prolongs the survival of cardiac retransplantation allografts in a mouse model

**DOI:** 10.3892/etm.2015.2204

**Published:** 2015-01-22

**Authors:** TENG MA, JIACHENG XU, JIAWEI ZHUANG, XIAOBIAO ZHOU, LIANFENG LIN, ZHONGGUI SHAN, ZHONGQUAN QI

**Affiliations:** 1 Department of Cardiac Surgery, The First Affiliated Hospital, Xiamen University, Xiamen, Fujian 361003, P.R. China; 2 Organ Transplantation Institute, Medical College, Xiamen University, Xiamen, Fujian 361005, P.R. China

**Keywords:** chemokines, cardiac retransplantation, C-X-C motif chemokine 9, C-X-C motif chemokine 10, FTY720

## Abstract

The upregulation of chemokine genes and the subsequent T-lymphocyte recruitment to the graft are early events in the development of acute cardiac transplant rejection or cardiac allograft vasculopathy. In the present study, a combined immunosuppressive regimen of C-X-C motif chemokine 9 (CXCL9) antibody (Ab), CXCL10 Ab and FTY720 was used in order to reduce the infiltration of memory T lymphocytes and prolong graft survival in a retransplantation murine model. BALB/c donor hearts were transplanted heterotopically into C57BL/6 mice at day 28 after skin transplantation. The mice were divided into four groups: i) Control (normal saline), ii) CXCL9 Ab and CXCL10 Ab [150 μg; once daily (qd); intraperitoneal (ip)], iii) FTY720 (0.2 mg/day; qd; ip) and iv) combined (2 mg/kg/day; qd; ip). Measurements of the median survival time of the cardiac grafts, histological examination, reverse transcription-quantitative polymerase chain reaction analysis, enzyme-linked immunosorbent assay and a mixed lymphocyte reaction were performed. The median graft survival time of the combined group was prolonged (9.3 days) compared with that of the control group (3.5 days) (P<0.001). Histological examination revealed that the combined treatment group graft rejection pathological score was 0.50, while the control group score was 3.62 (P<0.001). In addition, the gene expression level of interleukin (IL)-2 was significantly lower and the levels of IL-10 and transforming growth factor-β (TGF-β) were significantly higher in the combined group compared with those in the control group (P<0.001). Furthermore, the serum concentration levels of IL-2 and interferon-γ (IFN-γ) were significantly lower (P<0.001) and the concentration of IL-10 was significantly higher (P<0.05) in the combined group compared with those in the control group. In the mixed lymphocyte reaction, T-cell proliferation was found to be significantly lower in the combined treatment group than that in the control group (P<0.001). In conclusion, treatment with CXCL9 Ab and CXCL10 Ab or FTY720 reduced the graft infiltration of inflammatory cells, inhibited T-cell proliferation and prolonged graft survival. The combined treatment regimen of CXCL9 Ab, CXCL10 Ab and FTY720 was found to significantly reduce the infiltration of inflammatory cells in the graft and prolong graft survival.

## Introduction

Cardiac transplantation is a viable therapy for patients with end-stage heart disease. The development of novel immunosuppressive agents has improved early survival following cardiac transplantation; however, long-term survival has linearly decreased (1). A second transplantation surgery is inevitable if previous grafts are completely rejected (2). Furthermore, a number of factors, including the initial transplant graft recipient, blood transfusion, pregnancy and continued exposure to symbiotic microbial pathogens, may stimulate memory T (Tm) cell proliferation; 40–50% of T cells express a memory-like phenotype in peripheral blood (3,4). Tm cells have become an inevitable barrier in transplant tolerance in retransplantation due to their ability to respond more rapidly and effectively to the previously encountered pathogens and their longevity (5).

Heart retransplantation studies face difficulties in establishing a retransplantation murine model, particularly a model of solid organ retransplantation with blood vessel anastomosis, since few mice can withstand a second surgical trauma. In the present study, an advanced microsurgical technique was used to adapt a consecutive skin-heart retransplantation murine model. As a potent immunogen, alloskin has the ability to vigorously induce alloantigen-specific Tm cells, resulting in a reliable retransplantation murine model.

Cardiac allograft vasculopathy (CAV) is the major cause of late morbidity and mortality among heart retransplantation recipients. Progressive neointimal proliferation leads to ischemic injury and failure of the allograft (6). Although the pathogenesis of CAV is not fully understood, a number of studies have found that the infiltration of T cells (including naive T cells and Tm cells) and their expression products is a distinctive characteristic of CAV (7,8). Researchers have attempted to investigate immunosuppressive regimens for Tm cells from multiple perspectives; however, previous studies have indicated that conventional immunosuppressive regimens, such as antirejection drugs (including cyclosporin A, FK506 and rapamycin) and classic costimulatory pathway blockade are ineffective in retransplantation (9,10).

Chemokines are a family of ~50 cytokines that mediate cell chemotaxis and activation. Chemokine levels are known to significantly increase during acute rejection episodes following first organ transplantations (11,12). A previous study indicated that specific chemokines may play a critical role in Tm-cell recruitment during transplantation allograft rejection (13). We previously demonstrated the presence of higher levels of RANTES expression and secretion in retransplantation or Tm-cell-transfer models (14). In the present study, the effects of mouse C-X-C motif chemokine 9 (CXCL9) affinity purified polyclonal antibody (Ab), mouse CXCL10 monoclonal Ab and FTY720 on Tm-cell infiltration and allograft survival time were investigated in a murine retransplantation model.

## Materials and methods

### Animals

BALB/c (H2^d^) and C57BL/6 (H2^b^) mice were used as transplant donors and recipients, respectively. The mice (female; age, 8–12 weeks; weight, 20–25 g) were purchased from the Shanghai Laboratory Animal Center, CAS (Shanghai, China) and housed in the animal facilities at Xiamen University (Xiamen, China) under pathogen-free conditions. The animals received human care in compliance with the Guide for the Care and Use of Laboratory Animals published by the United States Department of Health and Human Services (8th edition, National Academies Press, Washington, USA, D.C., 2011). The current study was approved by the Ethics Committee of the First Affiliated Hospital of Xiamen University (Xiamen, China).

### Skin transplantation

Orthotopic full-thickness skin grafts were obtained from the BALB/c donors, cut into circular pieces (area, 1.5 cm^2^) and sutured bilaterally into the flanks of the C57BL/6 recipients. The BALB/c donor skin grafts on the lumbar region of the C57BL/6 recipients were examined daily.

### Heterotopic heart transplantation and Ab treatment

Mouse hearts were transplanted to a heterotopic neck location by anastomosis of the neck vessels using a microsurgery nonsuture cuff technique (15). The BALB/c (H2^d^) mice were used as heart transplant donors, while C57BL/6 (H2^b^) mice received the heart transplants four weeks after the skin transplantation. Graft viability was assessed by palpation twice a day. The post-transplantation treatment regimens are shown in Table I.

The mice were divided into four groups: i) Control (n=6), ii) CXCL9 Ab and CXCL10 Ab (n=6), iii) FTY720 (n=6) and iv) combined (n=6). Mouse polyclonal CXCL9 Ab and mouse monoclonal CXCL10 Ab (cat. nos. AF-492-NA and MAB66, respectively; R&D Systems, Inc., Minneapolis, MN, USA) were administered [150 μg; intraperitoneal (ip)] to the transplant recipients on days −1, +1, +3 and every two days thereafter until rejection (the day of transplant counted as day 0). Similarly, FTY720 (R&D Systems, Inc.) was administered (0.2 mg; ip) to the transplant recipients on days −1, +1, +3 and every two days until rejection. Mice in the control group were treated with equivalent doses of normal saline. In the combined group, 150 μg CXCL9 Ab ip, 150 μg CXCL10 Ab ip and 0.2 mg FTY720 ip were administered to the transplant recipients on days 1, +1, +3 and every two days until rejection.

### Allograft survival time

The cardiac allografts were examined by palpation daily, at 8:00 a.m. and 5:00 p.m. Transplant rejection was defined as the absence of heartbeat, which was later confirmed by histological examination. Four groups were studied in total, containing 6 animals/group: positive control (n=6), CXCL9 Ab and CXCL10 Ab (n=6), FTY720 (n=6) and combined (n=6). All reagents were administered to the transplant recipients on days 1, +1, +3 and every 2 days thereafter until rejection. Mice in the control group were treated with equivalent doses of normal saline. The allograft survival time was recorded in each group.

### Histological examination

The cardiac allografts were harvested four days after transplantation and incised with a scalpel along the septal plane. Half of each sample was placed in 10% neutral buffered formalin (Shanghai HuaYi Bio-tech Co. Ltd., Shanghai, China) and embedded in paraffin (Shanghai HuaYi Bio-tech Co. Ltd.). Subsequently, 5-μm sections were cut, stained with hematoxylin and eosin (Shanghai HuaYi Bio-tech Co. Ltd.) and observed under an optical microscope (BM2000; Ronbio Scientific Co., Ltd., Shanghai, China). Allograft rejection level determination was performed according to the International Society for Heart and Lung Transplantation (ISHLT) standards (16,17).

### Reverse transcription-quantitative polymerase chain reaction (RT-qPCR)

Total RNA was extracted from the remainder of the cardiac allograft samples using TRIzol^®^ reagent (Invitrogen Life Technologies, Carlsbad, CA, USA), according to the manufacturer’s instructions. A total of 2 μg RNA was reverse-transcribed into cDNA using an ABI StepOnePlus™ system (Applied Biosystems Life Technologies, Foster City, CA, USA). RT-qPCR was performed using an MJ Research DNA Engine with a Chrome 4 Detector (PTC-200; MJ Research, Inc., St. Bruno, QC, Canada), with endogenous β-actin used as a reference. The gene expression levels of interleukin (IL)-2, interferon (IFN)-γ, IL-10 and transforming growth factor (TGF)-β were determined in each group. The primers used in this study (designed using Primer Premier 5.0 software; Premier Biosoft, Palo Alto, Canada) were as follows: β-actin, 5′-CATCCGTAAAGACCTCTATGCCAAC-3′ (forward) and 5′-ATGGAGCCACCGATCCACA-3′ (reverse); IFN-γ, 5′-CGGCACAGTCATTGAAAGCCTA-3′ (forward) and 5′-GTTGCTGATGGCCTGATTGTC-3′ (reverse); IL-2, 5′-GGAGCAGCTGTTGATGGACCTAC-3′ (forward) 5′-AATCCAGAACATGCCGCAGAG-3′ (reverse); IL-10, 5′-GACCAGCTGGACAACATACTGCTAA-3′ (forward) 5′-GATAAGGCTTGGCAACCCAAGTAA-3′ (reverse); and TGF-β, 5′-TGACGTCACTGGAGTTGTACGG-3′ (forward) and 5′-GGTTCATGTCATGGATGGTGC-3′ (reverse). The cycle conditions were as follows: 10 min denaturation at 95°C followed by a total of 40 cycles (95°C for 30 sec and 55°C for 1 min) and 1 min elongation at 72°C.

### Enzyme-linked immunosorbent assay (ELISA)

Blood was collected from the eyeballs of the recipient mice in the first four days after transplantation. The serum was separated by centrifugation (1,000 × g at 25°C for 10min), and the expression levels of IL-2, IFN-γ, IL-10 and TGF-β were determined using an ELISA kit (Shanghai Yikesai Bioproduct Co., Ltd., Shanghai, China). The serum optical density values were subsequently determined using an ELISA analyzer (iMark^™^; Bio-Rad Laboratories, Inc., Hercules, CA, USA), and the concentration of the cytokines was calculated using a standard curve.

### Mixed lymphocyte reaction

Spleen removal was performed in the C57BL/6 mice four weeks after skin transplantation. Lymphocytes were isolated from the spleen samples using an EZ-Sep™ lymphocyte separation kit (Dakewe Biotech Co., Ltd., Shenzhen, China) and the T cells were passed through nylon wool (Wako Pure Chemical Industries, Ltd., Osaka, Japan). The spleen cells of BALB/c mice were collected (4 weeks after transplant donation) and treated with 40 g/ml mitomycin (Amresco, Solon, OH, USA). The BALB/c and C57BL/6 cells were mixed at a ratio of 1:10 and cultured at 37°C for 72 h in triplicate. The degree of proliferation was measured in each group using a cell proliferation ELISA BrdU kit (Roche Applied Science, Mannheim, Germany).

### Statistical analysis

Data are presented as the mean ± standard error of the mean. SPSS 13.0 (SPSS Inc., Chicago, IL, USA) and GraphPad Prism 5 (GraphPad Software, San Diego, CA, USA) software were used to perfom the statistical analysis. Survival times were analyzed using the Kaplan-Meier method. All the animal group comparisons were evaluated using the Student’s t-test. P<0.05 and P<0.01 were considered to indicate a statistically significant difference.

## Results

### Allograft survival time

**Figure 1:**
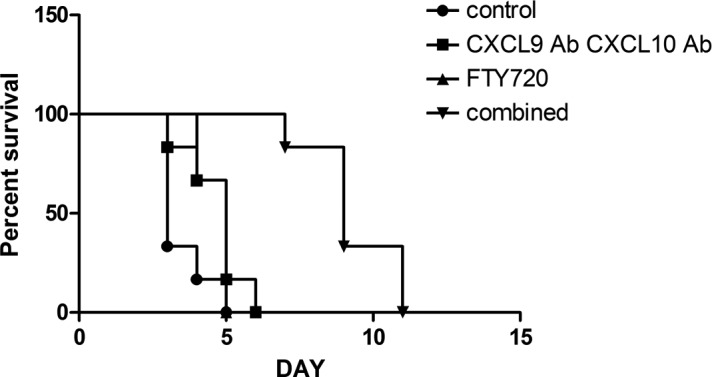
Cardiac allograft survival time in transplant recipient mice. C57BL/6 mice received BALB/c cardiac allografts and were divided into four groups: Control, CXCL9 Ab and CXCL10 Ab, FTY720, and combined (n=6 in each group). The graft median survival time was 3.5, 4.7, 4.7 and 9.3 days in each group, respectively. Combined group, CXCL9 Ab + CXCL10 Ab + FTY720; CXCL, C-X-C motif chemokine; Ab, antibody.

All the recipient mice survived the study, even following transplant rejection. In the murine heterotopic cardiac transplantation model, the mean graft survival time was found to be 3.5 days in the control group; the mean allograft survival time was prolonged in the CXCL9 Ab and CXCL10 Ab (4.7 days) and the FTY720 (4.7 days) groups (n=6; P<0.05 vs. the control group). Furthermore, the mean allograft survival time was significantly prolonged in the combined group (9.3 days) (n=6; P<0.01 vs. the control group) (Fig. 1). The group survival time comparisons were evaluated using the Kaplan-Meier method.

### Histological examination

**Figure 2:**
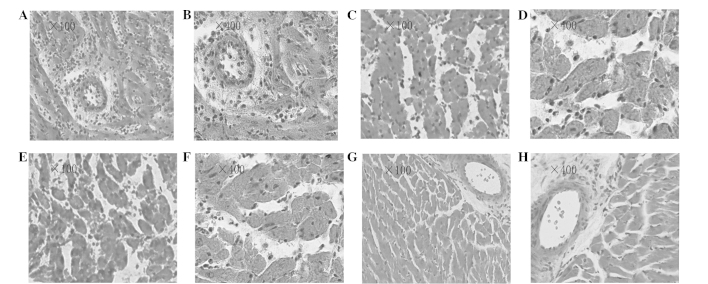
Graft pathological sections of each experimental group showing the rejection of cardiac allografts. On day 4 after heart transplantation, grafts were harvested and prepared for histological evaluation. (A and B) Control group at (A) ×100 and (B) ×400 magnification (ISHLT grade 4); (C and D) CXCL9 Ab and CXCL10 Ab group at (C) ×100 and (D) ×400 magnification (ISHLT grade 3A); (E and F) FTY720 group at (E) ×100 and (F) ×400 magnification (ISHLT grade 3B); and (G and H) combined group at (G) ×100 and (H) ×400 magnification (ISHLT grade 1A). Combined group, CXCL9 Ab + CXCL10 Ab + FTY720; ISHLT, International Society for Heart and Lung Transplantation; CXCL, C-X-C motif chemokine; Ab, antibody.

Heterotopic cardiac graft tissues were obtained on day 4 after transplantation. The heterotopic cardiac grafts in the control group showed severe acute rejection. Diffuse infiltration of various cell types was observed, along with edema, hemorrhage and extensive myocardial necrosis (Fig. 2A and B; ISHLT grade 4). By contrast, the CXCL9 Ab and CXCL10 Ab group grafts showed multifocal moderate acute rejection with multifocal inflammatory cell infiltration and myocardial necrosis (Fig. 2C and D; ISHLT grade 3A). The FTY720 group grafts showed diffuse severe acute rejection, with diffuse inflammatory cell infiltration and myocardial necrosis (Fig. 2E and F; ISHLT grade 3B). In the combined group, the grafts showed focal mild acute rejection. Focal myocardial interstitial and perivascular inflammatory cell infiltration was observed, with no evidence of myocardial necrosis (Fig. 2G and H; ISHLT grade 1A). Compared with the control group, graft rejection was alleviated to varying degrees in the treatment groups. The graft rejection pathology scores were 3.62±0.23 in the control group and 0.50±0.21 in the combined group; a statistically significant difference was observed between the two groups (P<0.01). By contrast, the graft rejection pathological scores were 2.50±0.20 in the CXCL9 Ab and CXCL10 Ab group and 2.62±0.23 in the FTY720 group; these scores were significantly different from the score in the control group (P<0.05).

### Allograft cytokine gene expression levels

**Figure 3:**
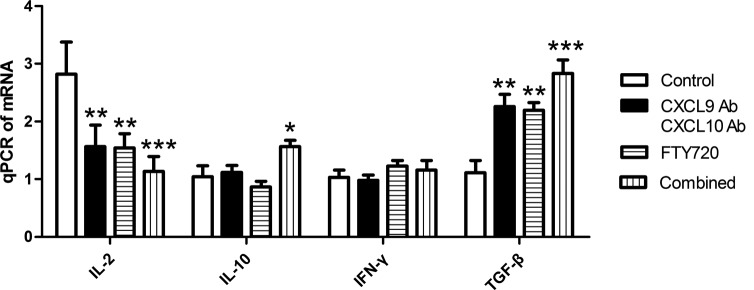
Relative gene expression levels of IL-2, IL-10, IFN-γ and TGF-β in the cardiac allografts of each group. Allograft mRNA was analyzed using qPCR. The gene expression level of IL-2 was significantly downregulated, while the gene expression level of TGF-β was significantly upregulated in the experimental groups compared with the control group. ^*^P<0.05, ^**^P<0.01 and ^***^P<0.001 vs. the control group. Combined group, CXCL9 Ab + CXCL10 Ab + FTY720; CXCL, C-X-C motif chemokine; Ab, antibody; IL, interleukin; IFN, interferon; TGF, transforming growth factor; qPCR, quantitative polymerase chain reaction.

Allograft mRNA was harvested from the heart samples obtained at day 4 post-transplantation. As shown in Fig. 3, the gene expression of IL-2 was downregulated, whereas the expression of IL-10 and TGF-β was upregulated. Statistical analysis revealed that IL-2 expression was significantly inhibited in the CXCL9 Ab and CXCL10 Ab and FTY720 groups compared with the control group (P<0.01), while the inhibition was even more marked in the combined group (P<0.001). By contrast, the gene expression of IL-10 was not affected by the CXCL9 Ab and CXCL10 Ab and FTY720 treatments, although it was upregulated in the combined treatment group. No statistically significant differences were observed in the gene expression of IFN-γ among the experimental groups. Compared with the control group, allografts in the CXCL9 Ab and CXCL10 Ab, FTY720 and combined groups showed varying degrees of TGF-β gene expression upregulation (P<0.05).

### Cytokine expression in peripheral blood

**Figure 4:**
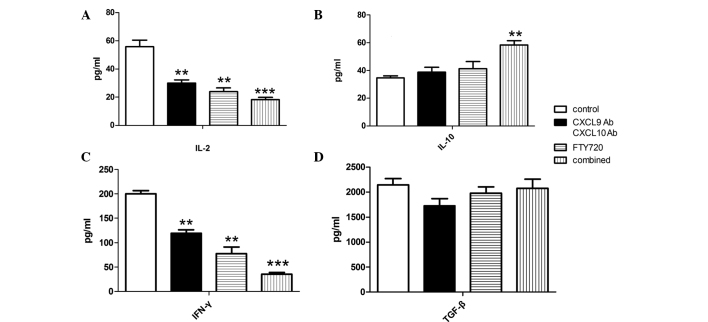
Serum concentration levels of (A) IL-2, (B) IL-10, (C) IFN-γ and (D) TGF-β, in the peripheral blood of recipient mice. The concentrations of IL-2 and IFN-γ were lower in the experimental groups than those in the control group. The concentration of IL-10 was higher in the serum of the combined experimental group than that in the control. ^**^P<0.01 and ^***^P<0.001 vs. the control group. Combined group, CXCL9 Ab + CXCL10 Ab + FTY720; CXCL, C-X-C motif chemokine; Ab, antibody; IL, interleukin; IFN, interferon; TGF, transforming growth factor.

To determine the cytokine levels in peripheral blood, serum was collected on day 4 after cardiac transplantation. Serum levels of IL-2, IL-10, IFN-γ and TGF-β were measured using ELISA, and the IL-2 and IFN-γ levels were found to be lower in the experimental groups than those in the control group. Statistical analysis revealed that the serum levels of IL-2 and IFN-γ in the CXCL9 Ab and CXCL10 Ab and FTY720 groups showed a decreasing trend (P<0.05) compared with those in the control group. Furthermore, the serum concentration of IL-2 and IFN-γ decreased more significantly in the combined group (P<0.001). As shown in Fig 4, administration of CXCL9 Ab and CXCL10 Ab or FTY720 alone did not affect the concentration of IL-10. However, IL-10 serum concentration was increased in the combined group (P<0.05). No statistically significant differences were observed in the TGF-β concentration among the groups (Fig. 4D).

### Comparison of mixed lymphocyte reaction

To assess the proliferative response differences among the groups, a mixed lymphocyte reaction was performed. Compared with the control group, the cell proliferative response was suppressed in the CXCL9 Ab and CXCL10 Ab and FTY720 groups (P<0.01). In addition, a significant difference in the proliferative response was observed in the combined group compared with the control group (P<0.001).

## Discussion

Acute cardiac allograft rejection is characterized by perivascular lymphocyte and monocyte infiltration; however, the exact mechanism leading to leukocyte recruitment in cardiac allografts has not been fully elucidated. A number of studies have indicated that Tm cells play an important role in this process (18,19). Allograft rejection mediated by Tm cells is resistant to conventional immunosuppressive treatments, such as administration of cyclosporin A, FK506 and rapamycin. Furthermore, conventional strategies and costimulatory blockades do not affect Tm cell-mediated rejection (20).

The role of chemokines in inflammatory processes has been studied extensively. The C-C chemokine receptor type 5 (CCR5)-binding chemokines CXCL9 and CXCL10 belong to the CXC family. It has previously been demonstrated that the expression levels of CXCL9, CXCL10 and CCR5 are widely upregulated in early retransplantation models (21). CXCL9 and CXCL10 bind the G-protein-coupled receptor C-X-C chemokine receptor type 5 (expressed on multiple cell types, but predominantly on memory-phenotype cells). Chemotaxis of inflammatory cells to inflammation sites activates Tm cells and accelerates the process of rejection. These observations indicate that CXCL9 and CXCL10 are closely associated with Tm cell-mediated retransplantation graft rejection (13); however, immunosuppressive treatment of secondary organ transplantation has been rarely reported.

**Figure 5:**
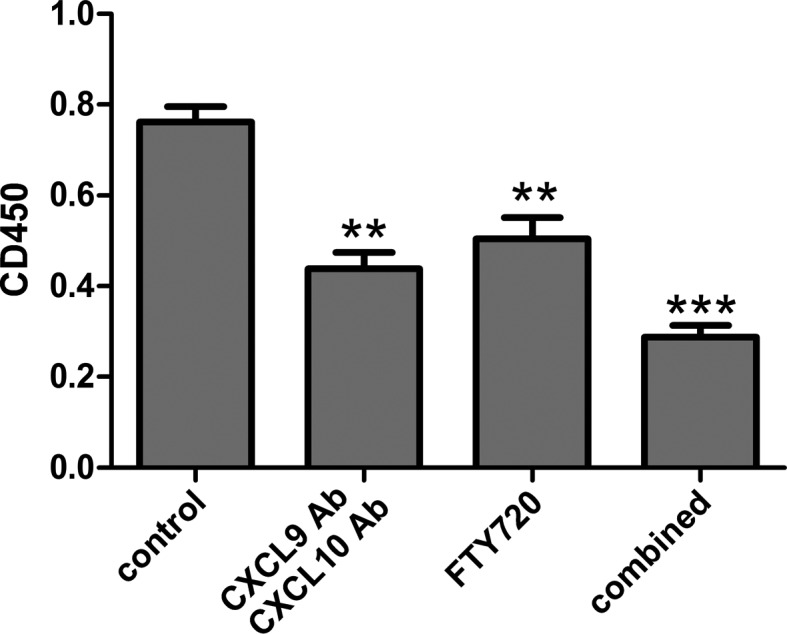
Effect of drug treatment on T-cell proliferation. The proliferative response in each group was assessed with the mixed lymphocyte reaction. ^**^P<0.01 and ^***^P<0.001 vs. the control group. Combined group, CXCL9 Ab + CXCL10 Ab + FTY720; CXCL, C-X-C motif chemokine; Ab, antibody; CD, cluster of differentiation.

In the present study, administration of CXCL9 Ab and CXCL10 Ab was shown to decrease the gene expression levels of the rejection cytokines IL-2 and IFN-γ in the graft and peripheral blood of transplant recipient mice. By contrast, the gene expression of the tolerance cytokines IL-10 and TGF-β was found to be upregulated (Figs. 3 and 4). The results indicated that CXCL9 Ab and CXCL10 Ab play an important role in the regulation of various cytokines secreted by Tm cells. The Tm-cell proliferation in the mixed lymphocyte reaction was also investigated. CXCL9 Ab and CXCL10 Ab administration was found to have an inhibitory effect on T-cell proliferation (Fig. 5). Histological examination revealed that CXCL9 Ab and CXCL10 Ab reduced the infiltration of lymphocytes in second organ retransplantation grafts (Fig. 2B and F). In addition, CXCL9 Ab and CXCL10 Ab treatment was shown to prolong the average survival time (Fig. 1). It was therefore demonstrated that CXCL9 Ab and CXCL10 Ab played an important role in Tm cell-mediated retransplantation graft rejection.

FTY720 is a novel potential immunosuppressant drug and is a form of *Cordyceps*, the active ingredient in Traditional Chinese Medicine. According to previous studies on FTY720, the drug induces lymphocyte homing to promote the apoptosis of lymphocytes and has an immunosuppressive role (22,23). FTY720 prompts the peripheral circulation to reduce the number of lymphocytes, reducing the lymphocyte infiltration of the graft or damaged tissue, thus delaying the rejection of the development process. Numerous studies have reported that the use of FTY720 prolongs graft survival in the skin, heart, liver and small bowel of animal transplantation models (24–27).

The results of the present study demonstrated that the use of FTY720 delayed the rejection of cardiac allografts (Fig. 1). The expression of TGF-β was upregulated in cardiac allografts, while the expression levels of IL-2 and IFN-γ were downregulated in the recipients’ peripheral blood (Figs. 3 and 4). Cytokine analysis revealed that FTY720 could regulate the cytokine levels in the grafts and peripheral blood. The mixed lymphocyte reaction results confirmed that FTY720 inhibited T-cell proliferation *in vitro* (Fig. 5), which is consistent with the results of Kim *et al* (28). In addition, histological examination of the FTY720 group revealed that FTY720 reduced the graft infiltration of lymphocytes.

In the present study, CXCL9 Ab, CXCL10 Ab and FTY720 were found to prolong cardiac allograft survival through various mechanisms. CXCL9 Ab and CXCL10 Ab prolong allograft survival by inhibiting the proliferation of activated Tm cells, whereas FTY720 prolongs allograft survival by accelerating lymphatic homing. The finding of the present study indicated that the application of CXCL9 Ab and CXCL10 Ab or FTY720 alone on Tm-cell-mediated second cardiac transplantation resulted in a certain inhibitory effect; however, the antirejection effect was not satisfactory. A combined blocking immunosuppressive regimen was therefore developed. It was hypothesized that combining the inhibitory effect of CXCL9 Ab and CXCL10 Ab on Tm-cell proliferation and the inducing effect of FTY720 on homing would efficiently prevent Tm and other inflammatory cells from migrating to the graft, and thus reduce or avoid the occurrence of acute rejection. In the combined group, the gene expression level of IL-2 in the graft was found to be significantly lower compared with that in the control group, while the gene expression level of TGF-β was found to be increased (Fig. 3). Furthermore, the expression levels of IL-2 and IFN-γ in the peripheral blood of the combined group were significantly lower than those in the control group, whereas the expression level of IL-10 was increased (Fig. 4). The combined group additionally showed the stronger inhibition of T-cell proliferation *in vitro* (Fig. 5). Pathological observations revealed that the combined application also significantly decreased the infiltration of lymphocytes in the grafts.

In conclusion, the results of the present study indicated that administration of CXCL9 Ab and CXCL10 Ab or FTY720 reduced the graft infiltration of inflammatory cells, inhibited T-cell proliferation and prolonged graft survival. Combined therapy with CXCL9 Ab, CXCL10 Ab and FTY720 was found to prolong the allograft survival by a considerably greater extent than single-drug therapy. The combined treatment may therefore be a novel therapeutic approach for the prevention of cardiac retransplantation graft failure.

## Figures and Tables

**Table I:** Post-transplantation treatment regimens.

Group	Treatment (intraperitoneal)
Control	NS
CXCL9 Ab and CXCL10 Ab	CXCL9 Ab + CXCL10 Ab + NS
FTY720	FTY720 + NS
Combined	CXCL9 Ab + CXCL10 Ab + FTY720 + NS

CXCL, C-X-C motif chemokine; Ab, antibody; NS, normal saline.

## References

[1] Taylor DO, Edwards LB (2006). Registry of the International Society for Heart and Lung Transplantation: twenty-third official adult heart transplantation report - 2006. J Heart Lung Transplant.

[2] Pour-Reza-Gholi F, Nafar M, Saeedinia A, Farrokhi F, Firouzan A, Simforoosh N, Basiri A, Einollahi B (2005). Kidney retransplantation in comparison with first kidney transplantation. Transplant Proc.

[3] Amir AL, D’Orsogna LJ, Roelen DL, van Loenen MM, Hagedoorn RS, de Boer R, van der Hoorn MA, Kester MG, Doxiadis II, Falkenburg JH (2010). Allo-HLA reactivity of virus-specific memory T cells is common. Blood.

[4] Bingaman AW, Farber DL (2004). Memory T cells in transplantation: generation, function, and potential role in rejection. Am J Transplant.

[5] Valujskikh A, Li XC (2007). Frontiers in nephrology: T cell memory as a barrier to transplant tolerance. J Am Soc Nephrol.

[6] Yun JJ, Whiting D, Fischbein MP, Banerji A, Irie Y, Stein D, Fishbein MC, Proudfoot AE, Laks H, Berliner JA, Ardehali A (2004). Combined blockade of the chemokine receptors CCR1 and CCR5 attenuates chronic rejection. Circulation.

[7] Iida S, Suzuki T, Tanabe K, Valujskikh A, Fairchild RL, Abe R (2013). Transient lymphopenia breaks costimulatory blockade-based peripheral tolerance and initiates cardiac allograft rejection. Am J Transplant.

[8] Wang H, Zhang Z, Tian W, Liu T, Han H, Garcia B, Li XC, Du C (2014). Memory T cells mediate cardiac allograft vasculopathy and are inactivated by anti-OX40L monoclonal antibody. Cardiovasc Drugs Ther.

[9] Liang H, Liao C, Qi Z, Sha C, Xie B, Chen J, Xia J, Wang Y, Yao Q, Zhao Y (2010). Rapamycin or tacrolimus alone fails to resist cardiac allograft accelerated rejection mediated by alloreactive CD4(+) memory T cells in mice. Transpl Immunol.

[10] Larsen CP, Knechtle SJ, Adams A, Pearson T, Kirk AD (2006). A new look at blockade of T-cell costimulation: a therapeutic strategy for long-term maintenance immunosuppression. Am J Transplant.

[11] Sallusto F, Schaerli P, Loetscher P, Schaniel C, Lenig D, Mackay CR, Qin S, Lanzavecchia A (1998). Rapid and coordinated switch in chemokine receptor expression during dendritic cell maturation. Eur J Immunol.

[12] Zhai Y, Wang Y, Wu Z, Kupiec-Weglinski JW (2007). Defective alloreactive CD8 T cell function and memory response in allograft recipients in the absence of CD4 help. J Immunol.

[13] Rosenblum JM, Shimoda N, Schenk AD, Zhang H, Kish DD, Keslar K, Farber JM, Fairchild RL (2010). CXC chemokine ligand (CXCL) 9 and CXCL10 are antagonistic costimulation molecules during the priming of alloreactive T cell effectors. J Immunol.

[14] Zhou X, Shan Z, Liang H (2013). Role of regulated upon activation normal T-cell expressed and secreted in a model of retransplantation acute rejection mediated by alloreactive memory CD4+T cells. Transplant Proc.

[15] Chen ZH (1991). A technique of cervical heterotopic heart transplantation in mice. Transplantation.

[16] Billingham ME, Cary NR, Hammond ME (1990). A working formulation for the standardization of nomenclature in the diagnosis of heart and lung rejection: Heart Rejection Study Group. The International Society for Heart Transplantation. J Heart Transplant.

[17] Winters GL, Marboe CC, Billingham ME (1998). The International Society for Heart and Lung Transplantation grading system for heart transplant biopsy specimens: clarification and commentary. J Heart Lung Transplant.

[18] Salom RN, Maguire JA, Hancock WW (1998). Endothelial activation and cytokine expression in human acute cardiac allograft rejection. Pathology.

[19] Setoguchi K, Hattori Y, Iida S, Baldwin WM, Fairchild RL (2013). Endogenous memory CD8 T cells are activated within cardiac allografts without mediating rejection. Am J Transplant.

[20] Farivar AS, Mackinnon-Patterson BC, McCourtie AS, Ward PA, Mulligan MS (2005). The role of CC and CXC chemokines in cardiac allograft rejection in rats. Exp Mol Pathol.

[21] Zhou X, Shan Z, Liang H, Lin Z, Qiu S, Kuang F, Zhuang J, Qi Z (2013). Role of regulated upon activation normal T-cell expressed and secreted in a model of retransplantation acute rejection mediated by alloreactive memory CD4^+^ T cells. Transplant Proc.

[22] Liu Y, Jiang J, Xiao H, Wang X (2012). Topical application of FTY720 and cyclosporin A prolong corneal graft survival in mice. Mol Vis.

[23] Heng Y, Ma Y, Yin H, Duan L (2010). Adoptive transfer of FTY720-treated immature BMDCs significantly prolonged cardiac allograft survival. Transpl Int.

[24] Pearl JP, Parris J, Hale DA, Hoffmann SC, Bernstein WB, McCoy KL, Swanson SJ, Mannon RB, Roederer M, Kirk AD (2005). Immunocompetent T-cells with a memory-like phenotype are the dominant cell type following antibody-mediated T-cell depletion. Am J Transplant.

[25] Zhang L, Zhu T, Sun EW, Shen SQ, Min ZL, Chen ZK (2003). Pretreatment with FTY720 alone induced long-term survival of mouse heart allograft. Transplant Proc.

[26] Wang ME, Tejpal N, Qu X, Yu J, Okamoto M, Stepkowski SM, Kahan BD (1998). Immunosuppressive effects of FTY720 alone or in combination with cyclosporine and/or sirolimus. Transplantation.

[27] Yanagawa Y, Hoshino Y, Chiba K (2000). The significance of timing of FTY720 administration on the immunosuppressive effect to prolong rat skin allograft survival. Int J Immunopharmacol.

[28] Kim MG, Lee SY, Ko YS, Lee HY, Jo SK, Cho WY, Kim HK (2011). CD4+ CD25+ regulatory T cells partially mediate the beneficial effects of FTY720, a sphingosine-1-phosphate analogue, during ischaemia/reperfusion-induced acute kidney injury. Nephrol Dial Transplant.

